# Supplementary material for: Venomix: a simple bioinformatic pipeline for identifying and characterizing toxin gene candidates from transcriptomic data
Source: PeerJ. 2018 Jul 31;6:e5361. doi: 10.7717/peerj.5361 (PMC6074769; doi:10.7717/peerj.5361)
Supplement: Supplemental Information 3 [file peerj-06-5361-s003.gz › FinalOutput_E-6/Vespryn_2/finaltree.pdf]

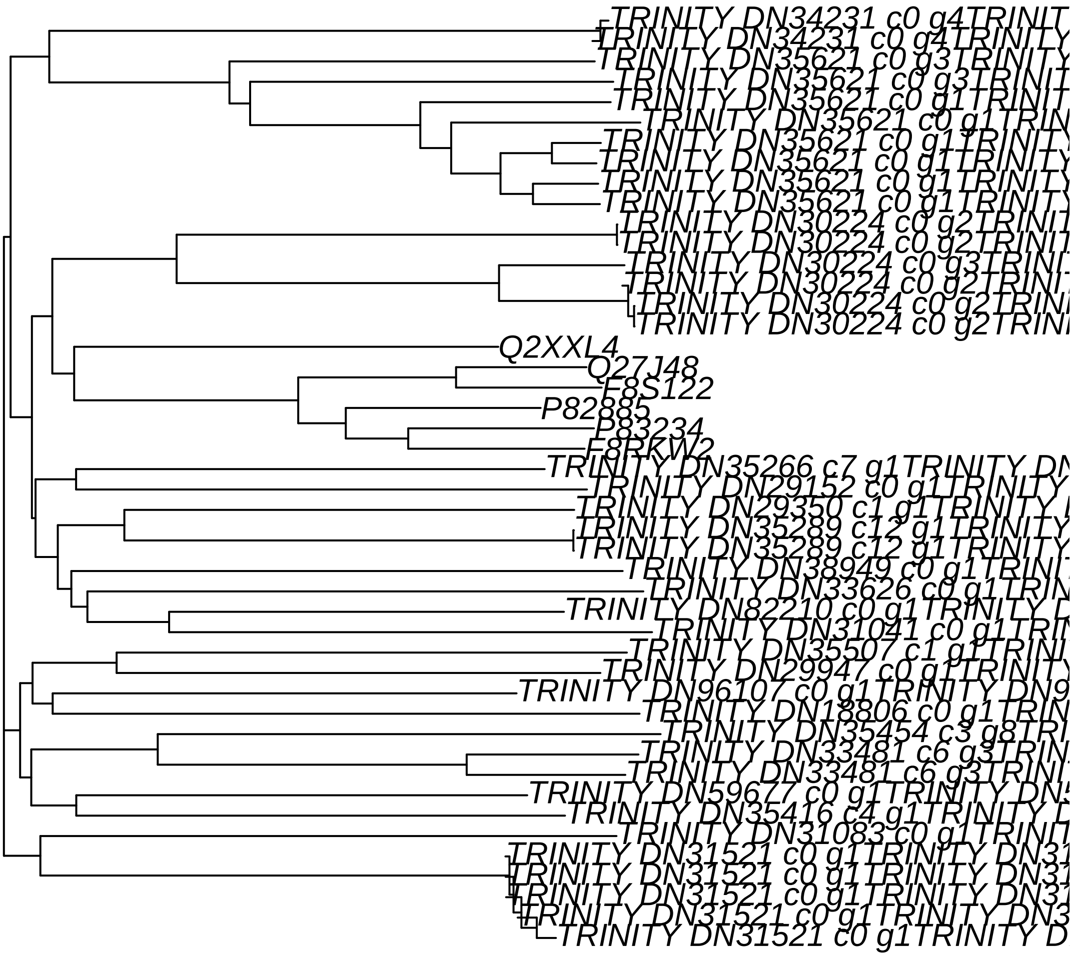
TRINITY DN34231 c0 g4TRINIT
TRINITY DN34231 c0 g4TRINITY
TRINITY DN35621 c0 g3TRINITY
TRINITY DN35621 c0 g3TRINIT
TRINITY DN35621 c0 g1TRINIT
TRINITY DN35621 c0 g1TRIN
TRINITY DN35621 c0 g1TRINIT
TRINITY DN35621 c0 g1TRINITY
TRINITY DN35621 c0 g1TRINITY
TRINITY DN35621 c0 g1TRINITY
TRINITY DN30224 c0 g2TRINIT
TRINITY DN30224 c0 g2TRINIT
TRINITY DN30224 c0 g3TRINI
TRINITY DN30224 c0 g2TRINI
TRINITY DN30224 c0 g2TRINI
TRINITY DN30224 c0 g2TRINI
Q2XXL4
Q27J48
F8S122
P82885
P83234
F8RKW2
TRINITY DN35266 c7 g1TRINITY DN
TRINITY DN29152 c0 g1TRINITY
TRINITY DN29350 c1 g1TRINITY
TRINITY DN35289 c12 g1TRINITY
TRINITY DN35289 c12 g1TRINITY
TRINITY DN38949 c0 g1TRINI
TRINITY DN33626 c0 g1TRIN
TRINITY DN82210 c0 g1TRINITY D
TRINITY DN31041 c0 g1TRIN
TRINITY DN35507 c1 g1TRIN
TRINITY DN29947 c0 g1TRINITY
TRINITY DN96107 c0 g1TRINITY DN9
TRINITY DN18806 c0 g1TRIN
TRINITY DN35454 c3 g8TRI
TRINITY DN33481 c6 g3TRIN
TRINITY DN33481 c6 g3TRINI
TRINITY DN59677 c0 g1TRINITY DN5
TRINITY DN35416 c4 g1TRINITY D
TRINITY DN31083 c0 g1TRINIT
TRINITY DN31521 c0 g1TRINITY DN3
TRINITY DN31521 c0 g1TRINITY DN3
TRINITY DN31521 c0 g1TRINITY DN3
TRINITY DN31521 c0 g1TRINITY DN3
TRINITY DN31521 c0 g1TRINITY D
